# The two-faced role of RNA methyltransferase METTL3 on cellular response to cisplatin in head and neck squamous cell carcinoma *in vitro* model

**DOI:** 10.3389/fonc.2024.1402126

**Published:** 2024-06-19

**Authors:** Kamila Ostrowska, Agnieszka A. Rawłuszko-Wieczorek, Julia Ostapowicz, Wiktoria M. Suchorska, Wojciech Golusiński

**Affiliations:** ^1^ Department of Head and Neck Surgery, Poznan University of Medical Sciences, Poznan, Poland; ^2^ Radiobiology Laboratory, The Greater Poland Cancer Centre, Poznan, Poland; ^3^ Department of Histology, Poznan University of Medical Sciences, Poznan, Poland; ^4^ Department of Electroradiology, Poznan University of Medical Sciences, Poznan, Poland

**Keywords:** RNA methylation, RNA methyltransferase-like 3, head and neck squamous cell carcinoma, chemotherapy, cisplatin

## Abstract

**Background:**

RNA methyltransferase-like 3 (METTL3) is responsible for methyl group transfer in the progression of *N* ^6^-methyladenosine (m^6^A) modification. This epigenetic feature contributes to the structural and functional regulation of RNA and consequently may promote tumorigenesis, tumor progression, and cellular response to anticancer treatment (chemo-, radio-, and immunotherapy). In head and neck squamous cell carcinoma (HNSCC), the commonly used chemotherapy is cisplatin. Unfortunately, cisplatin resistance is still a major cause of tumor relapse and patients’ death. Thus, this study aimed to investigate the role of METTL3 on cellular response to cisplatin in HNSCC *in vitro* models.

**Materials and methods:**

HNSCC cell lines (H103, FaDu, and Detroit-562) with stable METTL3 knockdown (sgMETTL3) established with CRISPR-Cas9 system were treated with 0.5 tolerable plasma level (TPL) and 1 TPL of cisplatin. Further, cell cycle distribution, apoptosis, CD44/CD133 surface marker expression, and cell’s ability to colony formation were analyzed in comparison to controls (cells transduced with control sgRNA).

**Results:**

The analyses of cell cycle distribution and apoptosis indicated a significantly higher percentage of cells with METTL3 knockdown 1) arrested in the G2/S phase and 2) characterized as a late apoptotic or death in comparison to control. The colony formation assay showed intensified inhibition of a single cell’s ability to grow into a colony in FaDu and Detroit-562 METTL3-deficient cells, while a higher colony number was observed in H103 METTL3 knockdown cells after cisplatin treatment. Also, METTL3 deficiency significantly increased cancer stem cell markers’ surface expression in all studied cell lines.

**Conclusion:**

Our findings highlight the significant influence of METTL3 on the cellular response to cisplatin, suggesting its potential as a promising therapeutic target for addressing cisplatin resistance in certain cases of HNSCC.

## 1 Introduction

RNA methylation occurring in the sixth position of adenosine [*N* ^6^-methyladenosine (m^6^A)] accounts for over 60% of all RNA modifications, particularly targeting mRNA and lncRNA but also microRNA, circRNA, rRNA, and tRNA (1, 2). Each mRNA typically contains approximately 3–5 m^6^A modifications located mainly near the stop codon, internal long exon, and 3′ untranslated region (3′ UTR) (3, 4). The m^6^A modification plays an important role in regulating gene expression by multiprotein complex cooperation known as “writers” that introduce the methyl group, “erasers” that remove them and determine the reversibility of the RNA methylation process, and “readers” that recognize and bind to methylated mRNA (5). The m^6^A methylase complex is composed of METTL3/14/16, RBM15/15B, ZC3H3, VIRMA, CBLL1, WTAP, and KIAA1429. The “erasers” consist of demethylases FTO and ALKBH5, while m^6^A binding proteins involve YTHDF1/2/3, YTHDC1/2 IGF2BP1/2/3, and HNRNPA2B1 (6, 7). As a post-transcriptional modification, RNA methylation regulates RNA splicing, nuclear export, stability, translation, DNA damage repair, initiation of miRNA biogenesis, and immunogenicity and, as a result, affects cellular differentiation, immune response, and the occurrence, development, and treatment response of cancer (8).

RNA methyltransferase-like 3 (METTL3) is identified as a predominant component responsible for the transfer of methyl group to the sixth position of adenosine (9). Methyltransferase activity of METTL3 can be detected in both the nucleus and cytoplasm, suggesting that METTL3 could modulate the metabolism and function of RNAs in various ways (10). Depending on the cancer type, the METTL3 may act as an oncogene or tumor suppressor (11). In most cases, METTL3 was reported as an oncogene to promote the initiation and development of cancers, including hematopoietic malignancies and solid tumors, through depositing m^6^A modification on critical transcripts (12–15). However, in renal cell carcinoma (RCC), higher expression of METTL3 may predict better patients’ survival outcomes possibly by promoting cell cycle arrest in the G1 phase and thus suppressing tumor growth (16). In the self-renewal glioblastoma stem cell (GSC), the knocking down of METTL3 significantly promoted tumor progression and shortened the lifespan of GSC-grafted animals (17). A similar conclusion was made for colorectal cancer (CRC), where METTL3 was found to suppress cell proliferation, migration, and invasion through p38/ERK pathways and thus supported patients’ longer survival time (18). To date, it was found that METTL3 overexpression promoted head and neck squamous cell carcinoma (HNSCC) cell proliferation, migration, invasion, and angiogenesis, while knockdown of METTL3 had the opposite effect *in vivo* and *in vitro* (19). Also, dysregulation of METTL3 significantly affects the total m^6^A methylation level (20) as we presented in our previous study based on HNSCC patients’ material, where METTL3 overexpression was positively correlated with high m^6^A modification level (21).

Given the research progress and interest in RNA methylation in regulating multiple biological processes, it is reasonable to speculate that m^6^A and METTL3 may also affect cellular response to chemotherapy (22). In HNSCC, cisplatin [*cis*-diamminedichloroplatinum(II) (CDDP)] is a commonly used chemotherapy that halts proliferation by inducing both cell cycle arrest and cell death. However, cisplatin resistance is a major cause of tumor relapse and patients’ death (23). The complexity of cisplatin resistance in HNSCC involves the occurrence of cancer stem cells, autophagy, epithelial–mesenchymal transition, drug efflux, and metabolic reprogramming (24). Due to the knowledge gap concerning METTL3’s impact on cisplatin response in HNSCC cells, we performed functional knockdown studies on an *in vitro* model and analyzed cell cycle distribution, apoptosis, expression of stem cell markers and the cells’ ability to colony formation after cisplatin treatment. As a result, we found that METTL3 deficiency may also sensitize HNSCC cells to cisplatin by more effective cell cycle arrest, apoptosis induction, and inhibition of colony formation and, in contrast, increased the expression of cancer stem cell markers.

## 2 Materials and methods

### 2.1 Cell culture

The FaDu and Detroit-562 cell lines were obtained from the American Type Culture Collection (ATCC™), while H103 was from the European Collection of Authenticated Cell Cultures (ECACC) (chosen cell lines correspond to different tumor locations: hypopharynx, metastatic pharynx, and tongue). The FaDu cells were cultured in Dulbecco’s modified Eagle’s Medium (DMEM) (Biowest, Nuaillé, France), the Detroit-562 cells in Eagle’s Minimum Essential Medium (EMEM) (Biowest, France), and the H103 cells in a 1:1 mixture of DMEM and Ham’s F12 Medium (Biowest, France). The 293T cell line (an epithelial-like cell obtained from the kidney) was used for lentiviral particle production and was cultured in DMEM. All growth media were supplemented with 10% fetal bovine serum (FBS; Biowest, France) and 1% penicillin/streptomycin (Biochrom, Holliston, MA, USA). The cell lines were cultured in an incubator at 37°C in a 5% CO_2_ atmosphere and at a humidity level of 95%.

### 2.2 Plasmid construction, transfection, transduction, and knockdown verification

**Figure 1:**
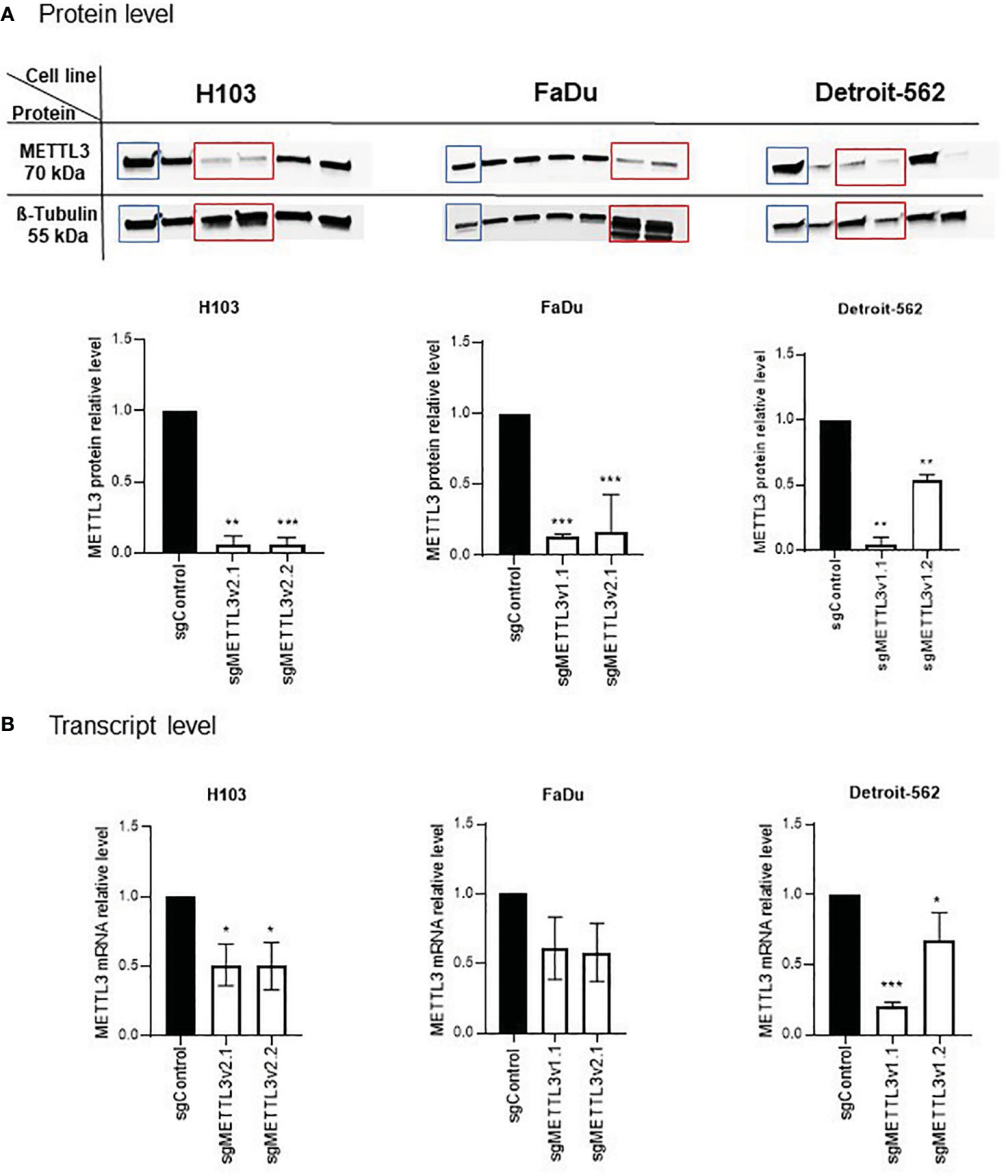
Validation of METTL3 knockdown by CRISPR-Cas9 in H103, FaDu, and Detroit-562 cell lines. Clonal selection of each cell line was performed to select the cells with statistically significant decreased levels of METTL3 at protein **(A)** and mRNA **(B)** levels in comparison to control sgRNA. Per cell line, two sgMETTL3 cell clones were selected (marked with red rectangle on protein blots). *p ≤ 0.05. **p < 0.01. ***p < 0.001.

LentiCRISPRv2 plasmid was a gift from Feng Zhang (Addgene plasmid #52961; http://n2t.net/addgene:52961; RRID: Addgene_52961) (25). METTL3 (sgRNA-1 and sgRNA-2) and control sgRNAs were designed based on the CRISPOR program (26), annealed, and cloned into the lentiCRISPRv2; the sgRNA oligonucleotide sequences are listed in **Supplementary Table S1**. Verification of proper sgRNA cloning was performed with Sanger sequencing. The resulting constructs were transduced into 293T cells with the packaging vector psPAX2 (Addgene #12260, USA) and envelope vector pMD2.G (Addgene #12259, USA) using the polyethylenimine (PEI) reagent. After 48 h of transduction, the supernatant containing viral particles was collected, filtered, and transferred into target cells supplemented with polybrene reagent (Sigma, St. Louis, MO, USA; TR1003, 10 μg/mL). After 48 h, cells were treated with the corresponding selective antibiotic puromycin (Sigma, P9620, 500 ng/mL) for 1 week to enrich modified cells. The clonal selection was performed to identify the cells with METTL3 knockdown. Thus, as a result for each cell line, two clones with different knockdown efficiency were selected for both designed METTL3 sgRNAs (hereinafter referred to as sgMETTL3v1.x or sgMETTL3v2.x, where x stands for clone number). Western blotting, qPCR, and RNA dot-blot were used to confirm the proper METTL3-deficient (sgMETTL3) cells’ selection (**Figure 1**; **Supplementary Figure 1**).

### 2.3 Western blotting analysis

Total protein was extracted using radioimmunoprecipitation assay (RIPA) buffer with the addition of protein inhibitors to extract proteins. The supernatant was used for further analysis. The proteins were separated in sodium dodecyl sulfate–polyacrylamide gel electrophoresis (SDS-PAGE) using Mini-PROTEAN TGX precast gels (Bio-Rad, Hercules, CA, USA). Subsequently, the gel was transferred to the polyvinylidene difluoride (PVDF) membrane using Trans-Blot Turbo transfer packs (Bio-Rad, Hercules, CA, USA), which was blocked with 5% milk in the TBST buffer for 1 h at room temperature. The membranes were incubated with primary antibodies against METTL3 (1:1,000; cat. no. PA5–121190; Invitrogen, Waltham, MA, USA) and β-tubulin (1:2,000; cat. no. PA5–16863; Invitrogen, Waltham, MA, USA) overnight at 4°C. Following the primary antibody incubation, membranes were incubated with a Rabbit horseradish peroxidase-conjugated secondary antibody (1:10,000; cat. no. NA934V; Cytiva, Marlborough, MA, USA) for 1 h at room temperature. Protein bands were visualized using Clarity Western ECL Blotting Substrate (Bio-Rad, Hercules, CA, USA) and ChemiDoc™ Touch Imaging System (Bio-Rad, Hercules, CA, USA). Protein expression was semi-quantified using ImageJ software (version 1.46; National Institutes of Health) with β-tubulin as the loading control.

### 2.4 RNA isolation and RT-qPCR

The RNA purification kit (RNeasy Mini Kit, Qiagen, Hilden, Germany) was used to extract total RNA from tissue specimens. The cDNA was synthesized using RevertAid First Strand cDNA Synthesis Kit (Thermo Fisher, Waltham, MA, USA) using 500 ng of total RNA, oligo dT primers, and random hexamer primers. The real-time quantitative polymerase chain reaction for individual gene expression analysis was conducted with a PowerTrack SYBR Green Master Mix (Thermo Fisher, Waltham, MA, USA) using the CFX96 Real-Time System (Bio-Rad, Hercules, CA, USA). The reaction conditions for all amplicons were as follows: initially, 95°C for 15 min, followed by 40 cycles at 95°C for 10 s, 60°C for 10 s, and 72°C for 10 s. The results were analyzed in triplicates by the 2^−ΔΔCt^ relative quantification method with GAPDH as a reference gene. The primer sequences used are listed in **Supplementary Table S1**.

### 2.5 m^6^A RNA dot-blot assay

The RNA dot-blot was completed for m^6^A to confirm the METTL3 knockdown effect. The isolated RNAs (final concentrations of 500 ng, 250 ng, and 25 ng) were incubated at 95°C in a heat block for 3 min to disrupt secondary structures and then chilled on ice immediately. The RNA solution was dropped on nitrocellulose membranes (Thermo Fisher Scientific, Shanghai, China) and UV-crosslinked. The membrane was blocked with 5% milk in TBST for 1 h, then incubated with primary m^6^A Rabbit Polyclonal Antibody (cat. no. MA5–35350, Invitrogen, Waltham, MA, USA) overnight at 4°C, and incubated with the Goat anti-Rabbit IgG Alexa Fluor 488 secondary antibody (cat. no. A-11008 Invitrogen, Waltham, MA, USA) for 1 h at room temperature. The immunoreactive dots were visualized using the ChemiDoc™ Touch Imaging System (Bio-Rad, Hercules, CA, USA).

### 2.6 Immunofluorescence

The immunofluorescence was completed for m^6^A to confirm the METTL3 knockdown effect. Cells were seeded on 8-well chamber slides (VWR, Darmstadt, Germany) with a density of 20,000 cells/well. After 48 h, the cells were washed with phosphate-buffered saline (PBS), fixed in 4% paraformaldehyde for 20 min at room temperature (RT), and permeabilized with ice-cold 100% methanol at −20°C for 20 min. Next, the blocking was performed by incubation with 0.2% Triton X-100 and 1% bovine serum albumin (BSA) (VWR, Germany) solution for 30 min at RT. After blocking, cells were washed with PBS. Next, 200 μL of a primary m^6^A antibody (cat. no. MA5–35350 Invitrogen, Waltham, MA, USA) was added into each chamber, and slides were incubated overnight at 4°C. After incubation, the cells were washed thrice with 2% BSA in PBS solution and incubated with 250 μL of Goat anti-Rabbit IgG Alexa Fluor 488 secondary antibody (cat. no. A-11008 Invitrogen, Waltham, MA, USA) for 1 h at 37°C in darkness. All slides were washed thrice with 2% BSA in PBS solution, and 400 μL of 4′,6-diamidino-2-phenylindole (DAPI) (cat. no. SAFSD8417 VWR, Germany) solution was added. Immunofluorescence was imaged using an Olympus IX83 microscope (Boston Industries, Inc., Walpole, MA, USA).

### 2.7 Flow cytometric analysis of cell cycle, apoptosis, and CD44+/CD133+ stemness marker expression

The cells (2 × 10^5^/well in 6-well plates) were treated with 1 tolerable plasma level (TPL), 0.5 TPL, and 0 TPL of cisplatin for 72 h in triplicates. To investigate the distribution of cell cycle phases, cells were stained with propidium iodide (20 μg/mL) (Cayman Chemical, Ann Arbor, MI, USA) and RNAse I (500 μg/mL) (Panreac AppliChem, Darmstadt, Germany) for 1 h at 37°C. To determine cell apoptosis (exhibiting live, early, late apoptotic, and death), Annexin-V (eBioscience anti-human Annexin-V/FITC kit, cat. no BMS147FI, Invitrogen, Linz, Austria) staining was used according to the manufacturer’s protocol. For stemness marker expression analysis, the cells were treated with CD44+ (APC; cat no. 1A-221-T100; EXBIO, Vestec, Czech Republic) and CD133+ (PE; cat no. 1P-819-T100; EXBIO, Czech Republic) antibodies for 30 min at 4°C. The FlowJo software V10 was then used to analyze the percentages of 1) cell cycle distribution, 2) viable and apoptotic cells, and 3) the median fluorescence value of CD44+ and CD133+ expression. All experiments were performed in three independent technical and biological repeats.

### 2.8 Colony formation assay

The optimized number of cells was plated in six independent replicates on 6-well plates, and after 24 h, doses of 0 TPL, 0.5 TPL, and 1 TPL of cisplatin were added. The cells were incubated for 14 days with medium change every 2 days. To close the assay, the cells were fixated with denatured ethanol and stained roughly with 2 mL of Coomassie Blue solution (Merck Millipore Corporation, Darmstadt, Germany) for 30 min. The plates were then washed in warm water, dried, and photographed using the ChemiDoc Touch Bio-Rad system (Hercules, Clearwater, FL, USA). The ImageJ program was applied to complete an automatic colony counting.

### 2.9 Cytostatic

Cisplatin (Teva Pharmaceuticals, Warsaw, Poland) was used in this study at a TPL (1 TPL = 6.667 μM). For every test, the cells were first seeded in appropriate numbers on 6-well plates; after 24 h, the cisplatin in doses of 0 TPL (0 μM, control group), 0.5 TPL (3.33 μM), and 1 TPL (6.667 μM), prepared in the cells’ medium, was added.

### 2.10 Statistical analysis

The normality of the observed data distribution was assessed using the Shapiro–Wilk test. The one-way ANOVA was conducted for multiple comparisons. If Levene’s test indicated that the variances were not equal across the groups, the unequal variance t-test (Welch’s t-test) was implemented. To calculate the differences for a complex system (more than two groups), multiple-comparison procedures were used by applying Tukey’s *post-hoc* test. Statistical analysis was performed using the GraphPad Prism 9.0.1 software, and p < 0.05 was considered statistically significant. The setting of the p-value was *p ≤ 0.05, **p < 0.01, ***p < 0.001, and ****p < 0.0001.

## 3 Results

### 3.1 Assessment of METTL3 deficiency in H103, FaDu, and Detroit-562 HNSCC cell lines

Cells were subjected to an analysis of the METTL3 downregulation effect after clonal selection. A significant effect was observed in H103, FaDu, and Detroit-562 cells; two sgMETTL3 clones were selected per cell line (together for METTL3 sgRNA-1 and sgRNA-2, described as sgMETTL3v1 and sgMETTL3v2, respectively). METTL3 deficiency was measured at the protein (**Figure 1A**) and transcriptional (**Figure 1B**) levels. For H103, METTL3 reduction was achieved at a protein level by a mean of 94% for sgMETTL3v2.1 and sgMETTL3v2.2 while at mRNA levels of 51% and 50%, respectively. For FaDu, METTL3 reduction was achieved at a protein level assessed by a mean of 84% for sgMETTL3v2.1 and a mean of 87% for sgMETTL3v1.1 while at mRNA levels of 42% and 39%, respectively. For Detroit-562, METTL3 reduction was achieved at a protein level by a mean of 96% for sgMETTL3v1.1 and a mean of 46% for sgMETTL3v1.2 while at mRNA levels of 80% and 41%, respectively. Moreover, the m^6^A RNA dot-blot assay (**Supplementary Figure 1A**) and m^6^A immunostaining (**Supplementary Figure 1B**) confirm the lower abundance of m^6^A modification after METTL3 knockdown in all selected cell lines.

### 3.2 METTL3 deficiency sensitizes HNSCC cells to cisplatin by inducing cell cycle arrest and cell death

**Figure 2:**
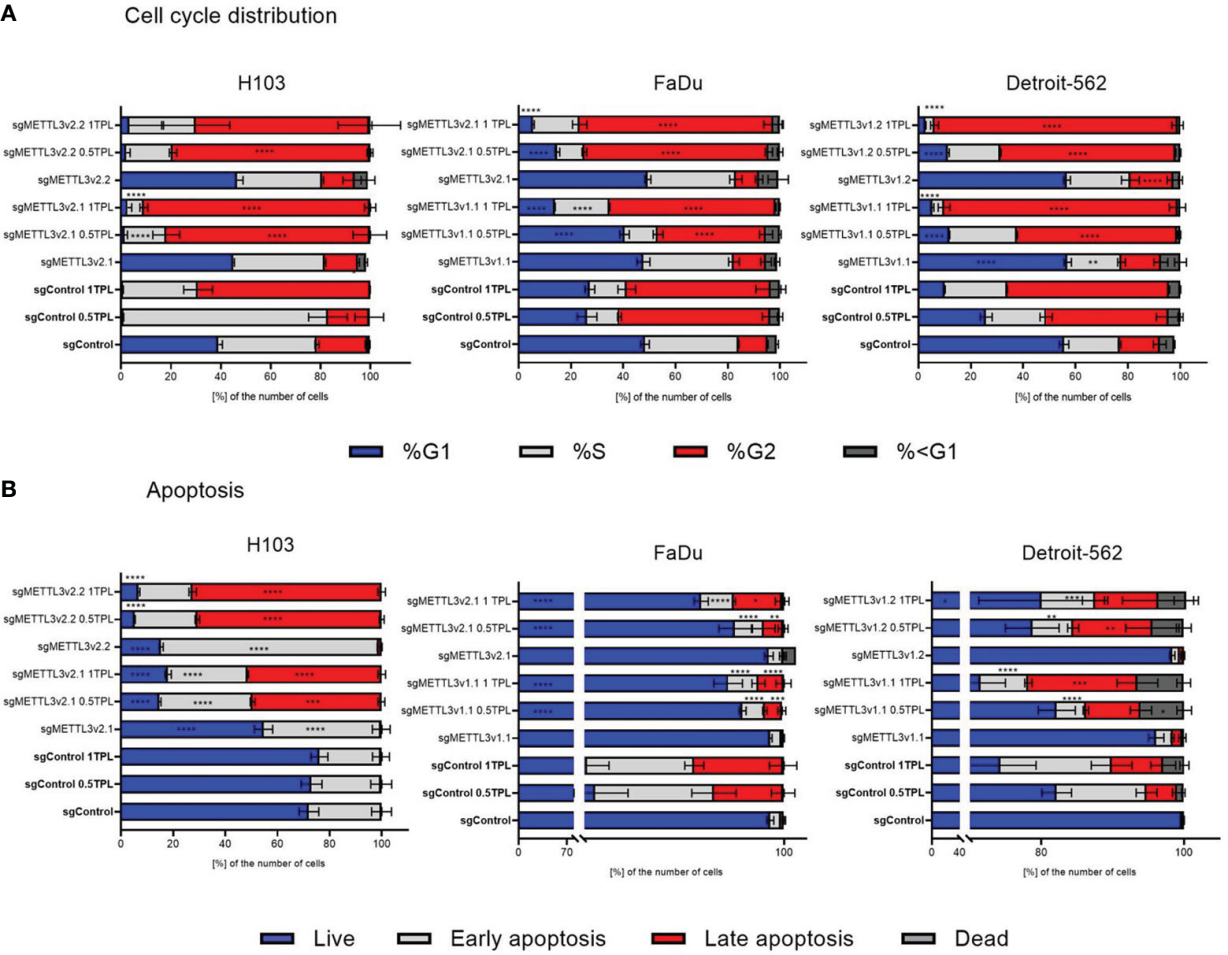
Flow cytometry detected the distribution of cell cycle phase **(A)** and apoptosis **(B)** after METTL3 knockdown in three independent cell lines—H103, FaDu, and Detroit-562—with different METTL3 knockdown efficiency and by three independent repeats. Each cell line was analyzed as two independent cell clones and control (transduced with control sgRNA). The cells were treated with doses of 0 TPL, 0.5 TPL, and 1 TPL of cisplatin for 72 h and analyzed. Data are represented as mean ± SD (n = 3) of the percentage number of cells; two-way ANOVA for multiple comparisons (here, we showed p-values for sgControl 0 TPL vs. all sgMETTL3 constructs 0 TPL; sgControl 0.5 TPL vs. all sgMETTL3 constructs 0.5 TPL; sgControl 1 TPL vs. all sgMETTL3 constructs 1 TPL). *p < 0.05. **p < 0.01. ***p < 0.001. ****p < 0.0001.

To investigate the METTL3 impact on cellular response to cisplatin, we established the stable METTL3-deficient (sgMETTL3) cell lines with CRISPR-Cas9. Further, we performed functional studies of cell cycle distribution and apoptosis with flow cytometry. Each cell line was divided into a cisplatin control group (0 TPL), and two experimental groups (doses of 0.5 TPL and 1 TPL of cisplatin). Further, we have performed multiple-comparison analysis for each cell line: sgControl 0 TPL vs. all sgMETTL3 0 TPL cells, sgControl 0.5 TPL vs. all sgMETTL3 0.5 TPL cells, and sgControl 1 TPL vs. all sgMETTL3 1 TPL cells. Our results indicate that lower METTL3 expression disrupted the cell cycle and apoptosis in three independent HNSCC cell lines—H103, FaDu, and Detroit-562—with two different METTL3 knockdown cell clones in three biological replicates (**Figure 2**). In cell cycle analysis, we observed the expected cisplatin effect (after both doses) by an increased percentage of cells in the G2 phase in all control cell types (transfected with control sgRNA) (**Figure 2A**). H103 and Detroit-562 cells with sgMETTL3 exhibited greater sensitivity to lower doses of cisplatin (0.5 TPL) when compared to sgControl cells. In the case of the FaDu cell line, both cisplatin doses caused similar results. However, we observed a statistically significant increased percentage of cells in the S and G2 phases after a 1-TPL dose of cisplatin treatment across all sgMETTL3 constructs in comparison to sgControl cells. In general, we observed a statistically significant higher percentage of cell arrest in the cell cycle in sgMETTL3 variants across all cell lines (**Figure 2A**). Moreover, cellular apoptosis analysis revealed cell sensitization to cisplatin for both sgMETTL3 variants in the H103 and Detroit-562 cell lines (**Figure 2B**). In contrast to Detroit-562, where cellular apoptosis exhibited a dose-dependent increase with higher cisplatin dose, the effect of cisplatin on H103 cells was dose-independent. Interestingly, we observed opposite results for the FaDu cell line that showed better cisplatin response in cells transfected with control sgRNA (**Figure 2B**). Flow cytometry data are available in **Supplementary Figure S3**.

### 3.3 METTL3 knockdown influences colony formation *in vitro*

**Figure 3:**
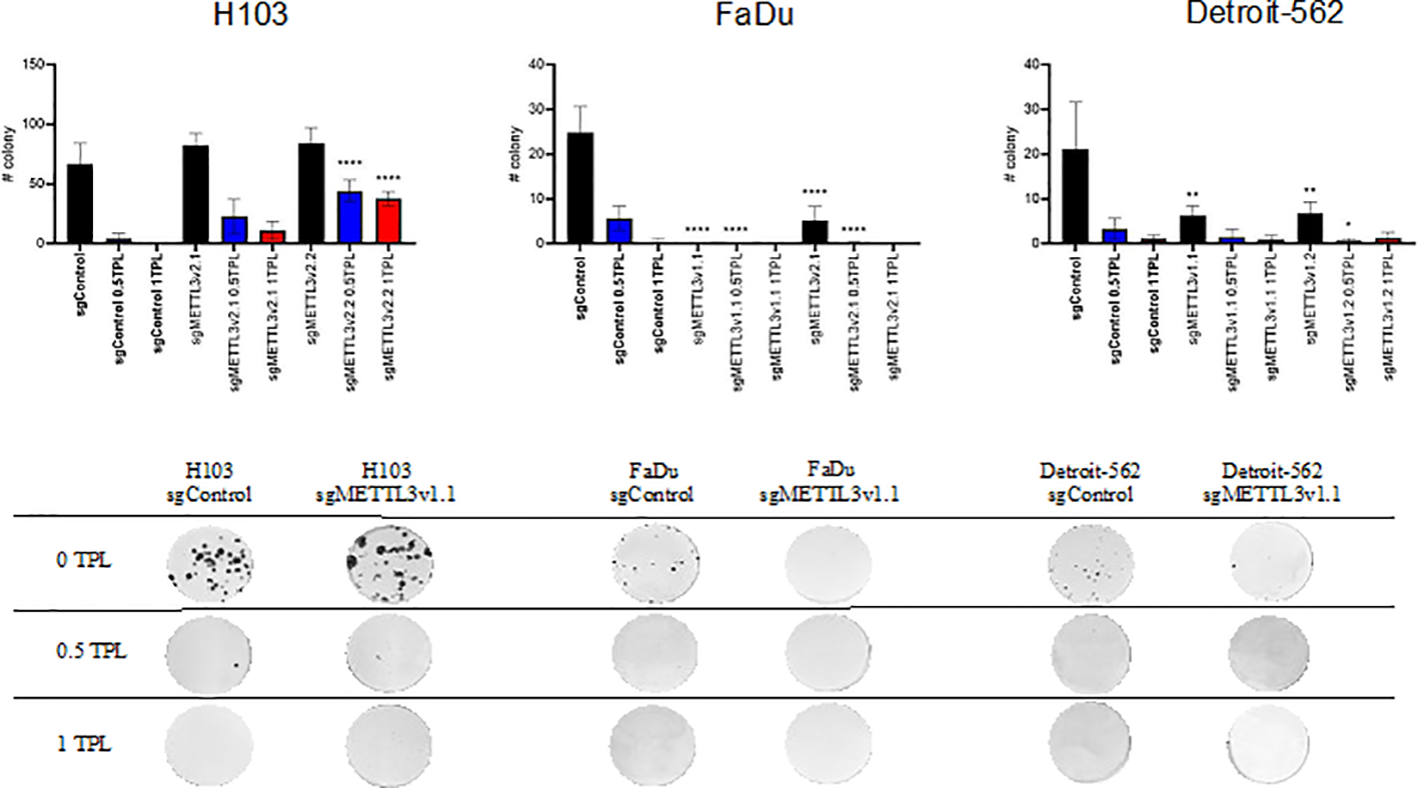
Colony formation assay analysis of three independent cell lines—H103, FaDu, and Detroit-562—with different METTL3 knockdown efficiency and by six independent repeats. Each cell line was analyzed as two independent cell clones and control (transduced with control sgRNA). The cells were treated with doses of 0 TPL, 0.5 TPL, and 1 TPL of cisplatin for 14 days and analyzed. Data are represented as mean ± SD (n = 6) number of colonies; ordinary one-way ANOVA comparison. *p < 0.05. **p < 0.01. ****p < 0.0001.

To elucidate if METTL3 deficiency may have an impact on the cells’ ability to create colonies in standard cultured conditions and after doses of 0.5 TPL and 1 TPL cisplatin treatment, we performed a colony formation assay. We used three independent cell lines—H103, FaDu, and Detroit-562—with various METTL3 knockdowns in six independent repeats. Overall, our observations revealed a consistent trend: as the concentration of cisplatin increased, the number of colonies decreased across both control (transfected with control sgRNA) and knockdown cells. Notably, for the 1-TPL cisplatin dose, the colony count was consistently less than 10 (**Figure 3**). Interestingly, the METTL3 knockdown significantly reduced the number of colonies in all studied variants of the FaDu and Detroit-562 cell lines. Consequently, a lower dose of cisplatin in these METTL3-deficient cells elicited effects comparable to those seen with higher doses in control cells. A similar trend was observed in the Detroit-562 cell line; however, cisplatin treatment of control and sgMETTL3 cell lines did not lead to significant differences in the number of colonies. For the H103 cell line, we obtained quite the opposite effect because METTL3 knockdown significantly enhanced the cells’ ability to colony formation. This resulted in markedly higher colony numbers across all studied variants and at all cisplatin doses compared to the control. Thus, we speculate that METTL3 may have diverse functions in tumor development depending on the location within head and neck cancer.

### 3.4 METTL3 knockdown increases extracellular CD44+ and CD133+ marker expression

**Figure 4:**
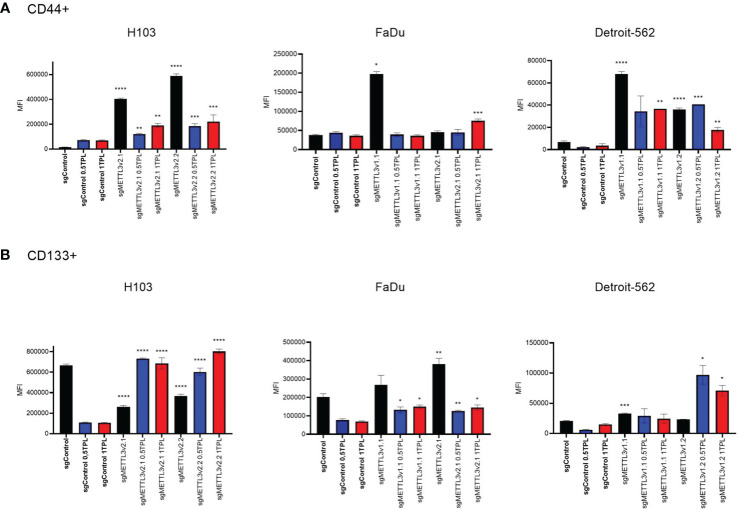
Flow cytometry analysis of CD44 **(A)** and CD133 **(B)** surface expression in three independent cell lines—H103, FaDu, and Detroit-562—with different METTL3 knockdown efficiency and by three independent repeats. Each cell line was analyzed as two independent cell clones and control (transduced with control sgRNA). The cells were treated with doses of 0 TPL, 0.5 TPL, and 1 TPL of cisplatin for 72 h and analyzed. Data are represented as mean ± SD (n = 3) median fluorescence intensity (MFI); ordinary one-way ANOVA comparison. *p < 0.05. **p < 0.01. ***p < 0.001. ****p < 0.0001.

Overexpression of cancer stem cell (CSC) markers contributes to tumor initiation, invasion, recurrence, and resistance to chemoradiotherapy including cisplatin treatment (24). CD44, a hyaluronic acid (HA) receptor, and CD133, also known as prominin-1, have been considered potential CSC markers in head and neck cancer. To investigate whether METTL3 influences the stemness properties of cells and potentially modulates their response to cisplatin, we conducted flow cytometric analysis to assess the expression levels of surface markers CD44 and CD133 in the H103, FaDu, and Detroit-562 cell lines with various sgMETTL3 cell clones in three independent biological repeats (**Figure 4**). Our study indicates that cisplatin treatment of control (transduced with control sgRNA) cells does not significantly influence CD44 expression in all studied cell lines. However, METTL3 knockdown increased the expression of CD44 in all cell lines and sgMETTL3 variants in comparison to sgControl. We observed a significant increase in CD44 in H103 sgMETTL3v2.1 and sgMETTL3v2.2 cells, and Detroit-562 sgMETTL3v1.1 and sgMETTL3v1.2 cells after both treatments of 0.5 TPL and 1 TPL cisplatin, and in FaDu sgMETTL3v2.1 cells after treatment 1 TPL cisplatin (**Figure 4A**). Similarly, the CD133 marker level is significantly increased in FaDu sgMETTL3V2.1 and Detroit-562 v1.1 sgMETTL3 cell lines, while H103 sgMETTL3 cells exhibited a lower level in comparison to sgControl cells (**Figure 2B**). Also, in all cell line controls, cisplatin treatment caused CD133 to decrease, the same as in FaDu sgMETTL3 cells (**Figure 4B**). In turn, in all studied cell lines, cisplatin leads to an increase in CD133 marker level (**Figure 4B**). Overall, our findings demonstrated elevated levels of surface CD44 and CD133 stemness markers in all sgMETTL3 cell lines following cisplatin treatment. Hence, we speculate that METTL3 may be a player in cancer cells’ cellular stemness-related process.

## 4 Discussion

As the most abundant internal mRNA modification in eukaryotic cells, m^6^A has emerged as an important regulator of gene expression and has a profound impact on cancer initiation and progression. mRNA m^6^A modification is regulated by m^6^A methyltransferases, demethylases, and reader proteins to fine-tune gene expression at the post-transcriptional level. The most well-studied m^6^A methyltransferase, METTL3, plays critical roles in modulating gene expression and influencing the malignant progression of various tumors. Its impact encompasses key aspects such as proliferation, invasion, metastasis, and drug resistance (27). Interestingly, METTL3 was found to act as an oncogene or a tumor suppressor. As an oncogene, METTL3 may introduce the methyl group into the mRNA of target genes such as *MYC* and *BCL2*, promoting its translation that leads to cell differentiation and proliferation and affects apoptosis in acute myeloid leukemia and breast cancer (12, 13). In liver cancer, METTL3 participates in RNA decay by facilitating the binding of m^6^A reader YTHDF2 to the *SOCS2* gene, ultimately promoting cancer cell proliferation and migration (14). Also, in hepatocellular carcinoma (HCC), METTL3 stabilizes LINC00958, Snail, and CTNNB1, thereby contributing to cellular processes such as lipogenesis, proliferation, metastasis, and tumor growth (28–30). In gastric cancer, m^6^A presence on lncRNA ARHGAP5-AS1 transcript leads to chemoresistance, while on LINC00470, it intensifies proliferation, migration, and invasion (31, 32). In lung cancer, *METTL3* overexpression facilitates drug resistance and metastasis by promoting the translation of YAP and MALAT1 transcripts in cooperation with YTHDF1/3 reader proteins (33). Moreover, in colorectal cancer, METTL3 targets genes such as *SOX2*, *HK2*, *SLC2A1*, and *CBX8*, inducing metastasis-related processes and activating the glycolysis pathway and cellular stemness (34–36). However, in renal cell carcinoma, METTL3 acts as a tumor suppressor. In RCC, it impacts the proliferation, migration, and apoptosis of cancer cells (16). Likewise, in endometrial cancer, METTL3 contributes to the decay of transcripts such as PHLPP2 and mTORC2 via YTHDF2, inhibiting cancer cell proliferation (37). Furthermore, in colorectal cancer, METTL3 overexpression has been associated with reduced cellular migration and invasion by affecting the p38/ERK pathway (18).

Our previous study highlighted the positive correlation between the abundance of total RNA m^6^A and the expression of selected methyltransferase (including METTL3), demethylase, and binding proteins in HNSCC tissues (21). These results were confirmed by other groups that found that m^6^A levels and METTL3 expressions in HNSCC tissues were significantly increased compared with paired adjacent normal tissues. Meanwhile, METTL3 emerged as an independent risk factor for the prognosis of HNSCC patients. Moreover, METTL3 overexpression promoted HNSCC cell proliferation, migration, invasion, and angiogenesis, while the knockdown of METTL3 had the opposite effect *in vivo* and *in vitro*. Mechanistically, METTL3 enhanced the m^6^A modification of CDC25B mRNA, thereby stabilizing it and upregulating its expression, consequently activating the G2/M phase of the cell cycle and driving HNSCC malignant progression (19). Also, in oral squamous cell carcinoma (OSCC), METTL3 was significantly upregulated in tissue samples and correlated with the poor prognosis of OSCC patients. Functionally, loss and gain studies illustrated that METTL3 promoted the proliferation, invasion, and migration of OSCC cells *in vitro* and that METTL3 knockdown inhibited tumor growth *in vivo* (38). The Cancer Genome Atlas (TCGA) database analyses of 502 HNSCC patients revealed that METTL3 and METTL14 mediated lncRNA LNCAROD overexpression, which was associated with advanced T stage and shortened patients’ overall survival. Moreover, depletion of LNCAROD attenuated cell proliferation and mobility *in vitro*, as well as tumorigenicity *in vivo*, whereas overexpression of LNCAROD exerted opposite effects (39).

Considering the critical importance of the RNA methylation process and the *METTL3* gene in cancer and the absence of well-defined molecular targets responsible for modulating the cellular response to cisplatin in HNSCC, here, we conducted functional studies of cell cycle distribution, apoptosis, CD44/CD133 surface marker expression, and cell’s ability to colony formation after cisplatin treatment *in vitro*. Due to the high genetic and histologic diversity of head and neck cancer, we employed three different established cell lines representing different tumor locations (tongue, hypopharynx, and metastatic pharynx). To investigate the METTL3 impact on analyzed cellular processes, we performed the METTL3 knockdown procedure with CRISPR-Cas9 (40). As a result, we obtained two clones for each cell line with the highest METTL3 knockdown efficiency. The differences in METTL3 deficiency across all studied cell lines are known as CRISPR-Cas9 drawbacks, including a lack of on-target editing efficiency, incomplete editing (mosaicism), and inaccurate on-target or off-target editing (41). Hence, the discrepancies among achieved data are the result of different sgRNA efficiency, and fold changes of selected features reflect the efficiency level of a given sgRNA. Our results indicate that METTL3 may be a genetic factor that both sensitizes and confers resistance to cisplatin in HNSCC. First, the analyses of cell cycle distribution and apoptosis revealed that a higher percentage of METTL3 knockdown cells was arrested in the G2/S phase and exhibited enhanced late apoptosis or death compared to control (cells transduced with control sgRNA). Additionally, the intensified inhibition of colony formation after cisplatin treatment may depend on METTL3 expression and tumor localization. Finally, we found that METTL3 may constitute a significant player in the acquisition of stemness potential by cancer cells. To the best of our knowledge, our study represents the first biological analyses of cellular response to cisplatin on three independent HNSCC cell lines with METTL3 knockdown. Our results are supported by the Qiao et al. study, which found that METTL3/METTL14 upregulation could enhance OSCC chemoresistance and accelerate tumor growth *in vivo* (42). To date, numerous studies conducted on patients’ material, as well as *in vitro*/*in vivo* functional and mechanistic studies, point out the significant role of the *METTL3* gene in various types of cancer, including HNSCC. However, there is still a lack of data indicating its involvement in treatment resistance-related processes, particularly regarding platinum derivatives that remain the mainstay systemic agents in solid tumors such as lung cancer, ovarian cancer, cervical cancer, and head and neck cancer. In HNSCC, cisplatin (CDDP) stands as a repeatedly confirmed gold standard systemic agent (43). Nonetheless, therapy resistance and subsequent near or distant relapses persist as common challenges, associated with high patient morbidity and a median survival of only 10 months (44, 45). The main biological processes and phenotypes implicated in the development of resistance mechanisms toward cisplatin in HNSCC encompass metabolic reprogramming, drug efflux, epithelial–mesenchymal transition, and the presence of cancer stem cells (24). Moreover, the acquisition of cisplatin resistance is linked to metabolic recovery from oxidative stress, dysregulated expression of genes involved in amino acid and fatty acid metabolism, central carbon catabolic pathways, enhanced glucose catabolism, and serine synthesis (46).

The significant limitations of our study include the lack of description of the molecular mechanisms and pathways underlying presented results, as well as specific targets for METTL3 action that could indicate the key genetic factors dependent on m^6^A RNA methylation and related to cisplatin response. Nevertheless, our study lays the groundwork for further, more comprehensive investigations into the role of the RNA methylation process in response to chemotherapy in HNSCC.

The multifaceted roles of METTL3 in regulating specific molecular signaling pathways across different types of cancers including head and neck cancer have been observed. Our study contributes to a better understanding of the METTL3 role in the cellular response to cisplatin, the gold standard treatment for HNSCC patients. Also, our findings underscore the significant METTL3 potential in regulating fundamental biological processes such as cell cycle, apoptosis, colony formation, and the acquisition of stemness characteristics in cancer cells, which profoundly impacts the efficiency of cisplatin treatment.

## Data availability statement

The original contributions presented in the study are included in the article/**Supplementary Material**. Further inquiries can be directed to the corresponding author.

## Author contributions

KO: Conceptualization, Formal analysis, Funding acquisition, Investigation, Methodology, Project administration, Visualization, Writing – original draft, Writing – review & editing. AR-W: Conceptualization, Writing – review & editing. JO: Methodology, Writing – review & editing. WS: Writing – review & editing. WG: Writing – review & editing.
