# Supplementary material for: The two-faced role of RNA methyltransferase METTL3 on cellular response to cisplatin in head and neck squamous cell carcinoma in vitro model
Source: Front Oncol. 2024 Jun 19;14:1402126. doi: 10.3389/fonc.2024.1402126 (PMC11223524; doi:10.3389/fonc.2024.1402126)
Supplement: Supplementary file 1 [file DataSheet_1.docx]

Supplementary Material

# Supplementary Figures and Tables

## Supplementary Figures


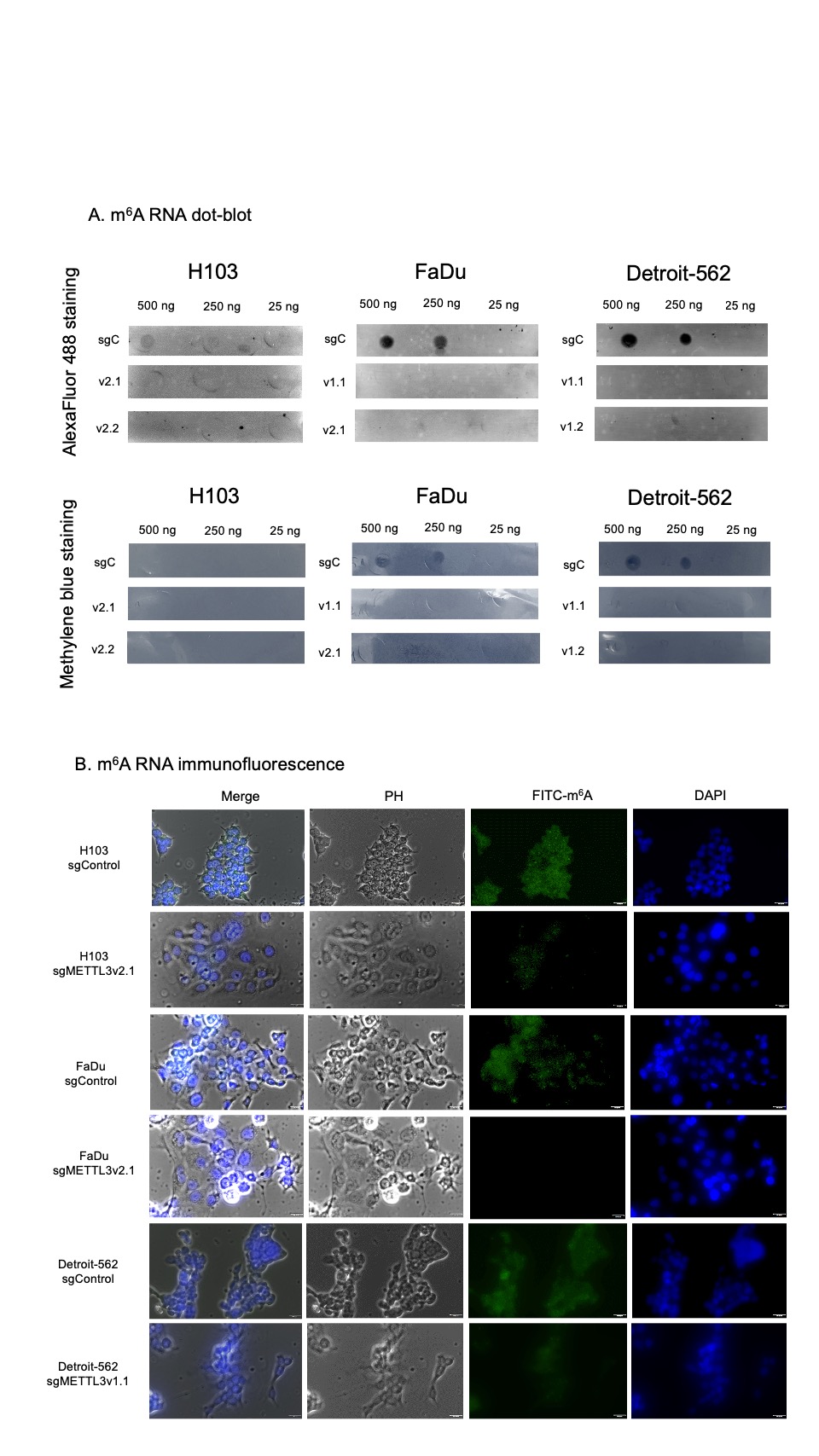


**Figure S1.** Validation of METTL3 knockdown by CRISPR/Cas9 in H103, FaDu, Detroit-562 cell lines by m^6^A RNA dot-blot AlexaFluor 488 and methylene blue staining (A) and m^6^A immunostaining (B). sgC- sgControl, v1.1-sgMETTL3v1.1, v1.2-sgMETTL3v1.2, v2.1-sgMETTL3v2.1, v2.2-sgMETTL3v2.2


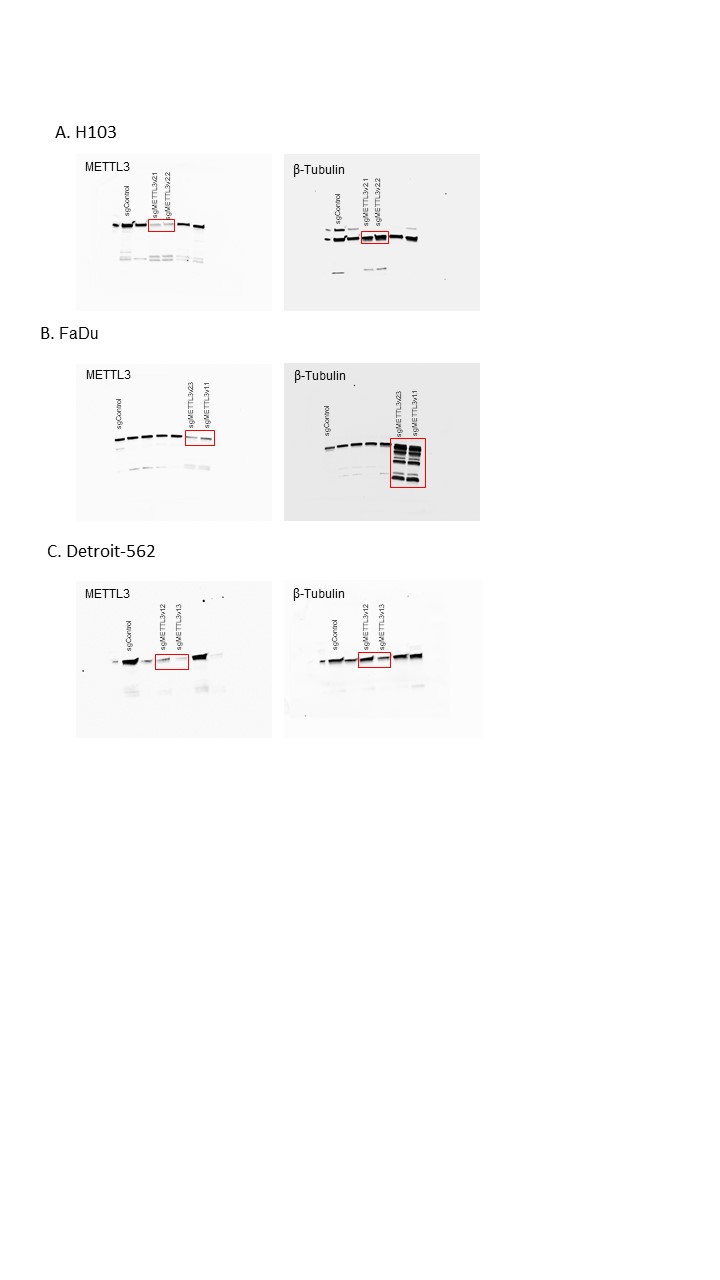


**Figure S2.** Western Blot validation of METTL3 knockdown by CRISPR/Cas9 in H103, FaDu, Detroit-562 cell lines. The samples marked with a red rectangle were selected for further analysis. METTL3 protein expression was semi-quantified using ImageJ software (version 1.46; National Institutes of Health) with β-tubulin as the loading control.


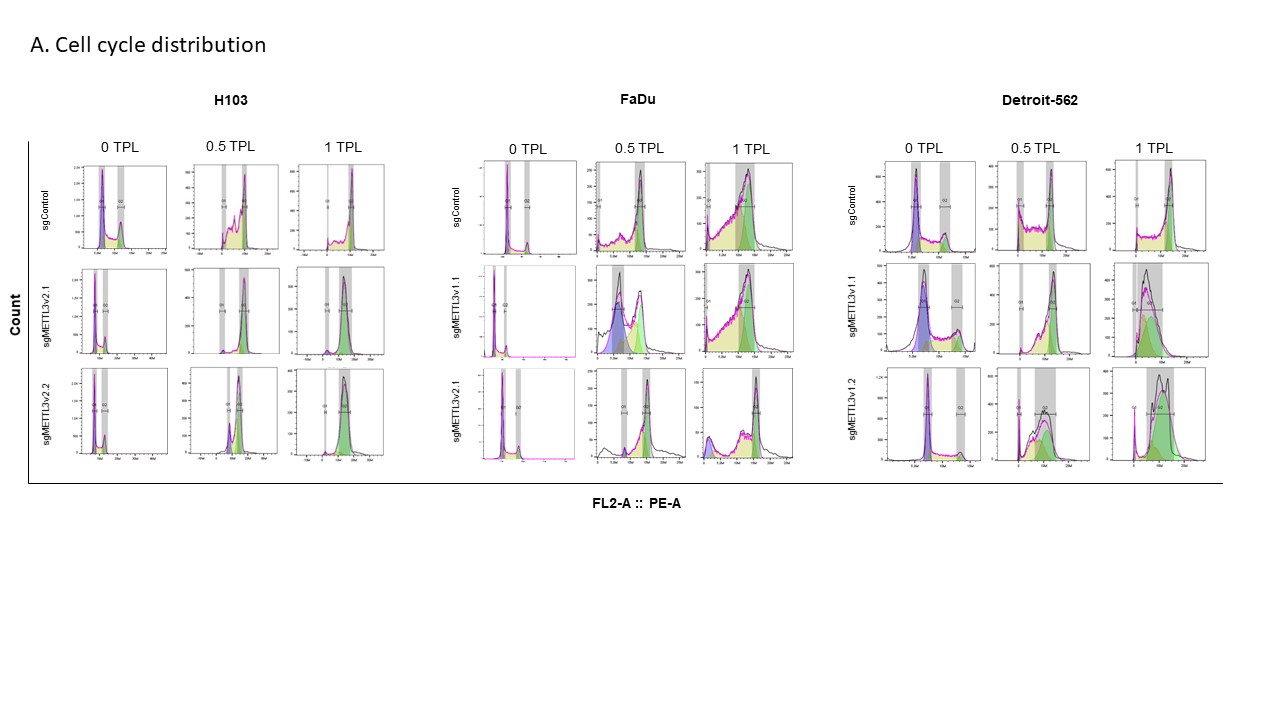


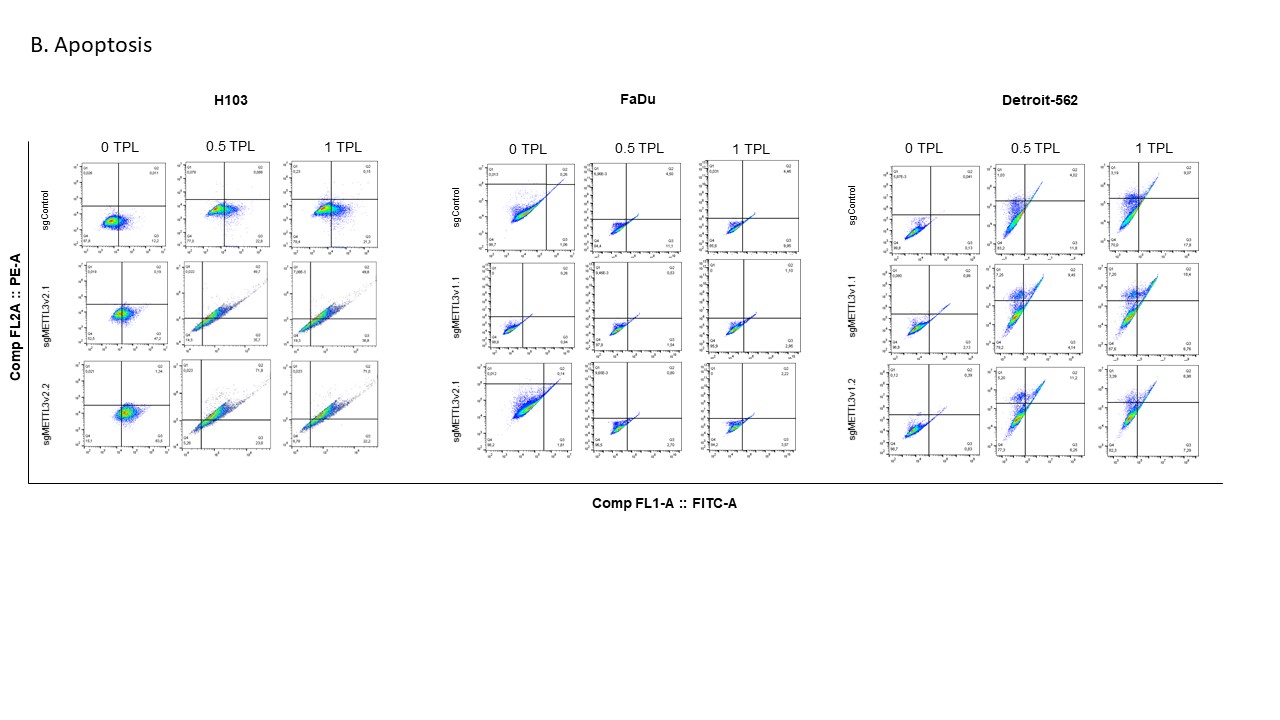


**Figure S3.** The flow cytometry analysis of cell cycle (A) and apoptosis (B) of H103, FaDu and Detroit-562 cell lines. Each cell line was analysed as a four two independent sgMETTL3 cell clones and control (transduced with control sgRNA). The cells were treated with 0TPL, 0.5TPL and 1TPL dosage of cisplatin for 72 h and analysed.

## Supplementary Tables

**Supplementary Table 1.** Oligonucleotide sequences

| **Gene** | **Type/Name** | **Sequence (5’-3’)** | **Genomic location (Assembly Dec.2023 GRCh38/hg38)** |
| --- | --- | --- | --- |
| METTL3 | gRNA-1  gRNA-2  PCR-fw  PCR-rev  SEQ-fw  SEQ-rev | TCCGCCGCGCCTTATTCGA  ACCTCTCGAATAAGGCGCG  ACAACAGAGCAAGAAGGTCAGT  GGGATTTCCTTTGACACCAACC  ATATCCTGGAGCGAGTGCTG  TCCACGTGTCCGACATCCTA | chr14: 21511248-21511267 (-)  chr14: 21511244-21511263 (+)  chr14: 21511279-21511301 (-)  chr14: 21511202-21511227 (-)  chr14: 21511279-21511298 (-)  chr14: 21511208-21511227 (+) |
| - | gRNA-control | AACCTAACGGGCTACGATACG | - |
| GAPDH | PCR-fw  PCR-rev | GTCTCCTCTGACTTCAACAGCG  ACCACCCTGTTGCTGTAGCCAA | chr12: 6537902-6537923 (+)  chr12: 6538115-6538136 (-) |
